# Malignant Phyllodes Breast Tumor: A Rare Case of Metastasis in Skeletal Muscle Detected on 18-Fluorodeoxyglucose Positron Emission Tomography

**DOI:** 10.7759/cureus.15274

**Published:** 2021-05-27

**Authors:** Ubaira Shaukat, Usama Rehman, Muhammad Numair Younis, Abubaker Shahid

**Affiliations:** 1 Nuclear Medicine, Institute of Nuclear Medicine & Oncology Lahore Cancer Hospital, Lahore, PAK; 2 Pathology, Shifa International Hospital Islamabad, Islamabad, PAK; 3 Radiation Oncology, Institute of Nuclear Medicine & Oncology Lahore Cancer Hospital, Lahore, PAK

**Keywords:** phyllodes tumors, fdg pet in phyllodes tumors, metastatic phyllodes tumors, rare metastasis of phyllodes breast tumor, skeletal muscles metastasis, fdg pet and skeletal muscle metastasis

## Abstract

Rapidly growing cystosarcoma phyllodes tumor (PT) of the breast are rarely encountered. Distant metastases are not uncommon in malignant PTs; however, rare sites of metastases are sometimes observed. Here, we present the case of a rapidly metastasizing malignant PT in which skeletal muscle metastasis was identified on 18F-fluorodeoxyglucose-based positron emission tomography-computed tomography reflecting its aggressive course and poor prognosis.

## Introduction

Phyllodes tumors (PTs) are very rare epithelial tumors, accounting for less than 1% of female breast neoplasms [1]. Women in their fourth and fifth decades are commonly affected by this disease [1]. These can be benign, borderline, or malignant depending on histological characteristics, such as margin involvement, cellularity, mitosis, and cellular atypia [1,2]. Distant metastasis and recurrence are rare among benign and borderline cases; however, they are not uncommon in malignant PT and range from 25% to 40% [3-5]. The tumor tends to metastasize via hematogenous spreading. The most common sites of involvement are bones, lungs, liver, and heart.

Skeletal muscle metastasis is extremely rare in malignant PT [1,5]. Surgery is the preferred treatment for PT [1]. Restaging of malignant tumors after treatment is vital for the assessment of local residual disease and the presence of distant metastases. 18F-fluorodeoxyglucose (FDG) positron emission tomography-computed tomography (PET-CT), which is a whole-body, sensitive, and metabolic imaging, is very useful for the assessment of residual lesions and evidence of distant metastases [6,7]. Here, we report a rapidly metastasizing case of malignant PT of the breast with rapid progression in a short period in which FDG PET-CT was used for restaging.

## Case presentation

**Figure 1:**
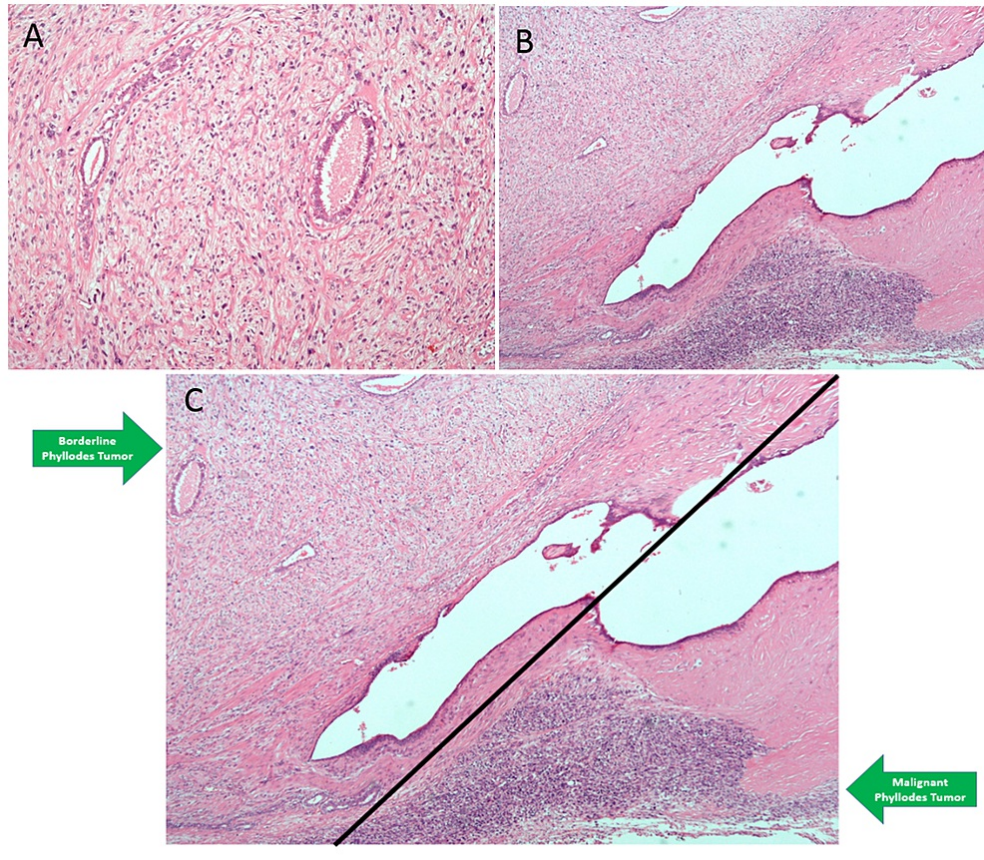
Histopathology of the excised breast lump. (A) Borderline phyllodes tumor (×100). (B) High-power view of many atypical stromal cells with a marked increase in cellularity, pronounced nuclear atypia, and several bizarre mitoses (×100). (C) Side-by-side areas of borderline and malignant phyllodes tumor. Borderline areas show epithelial rests and structures with stromal overgrowth. Total loss of epithelial elements is seen in the malignant component.

**Figure 2:**
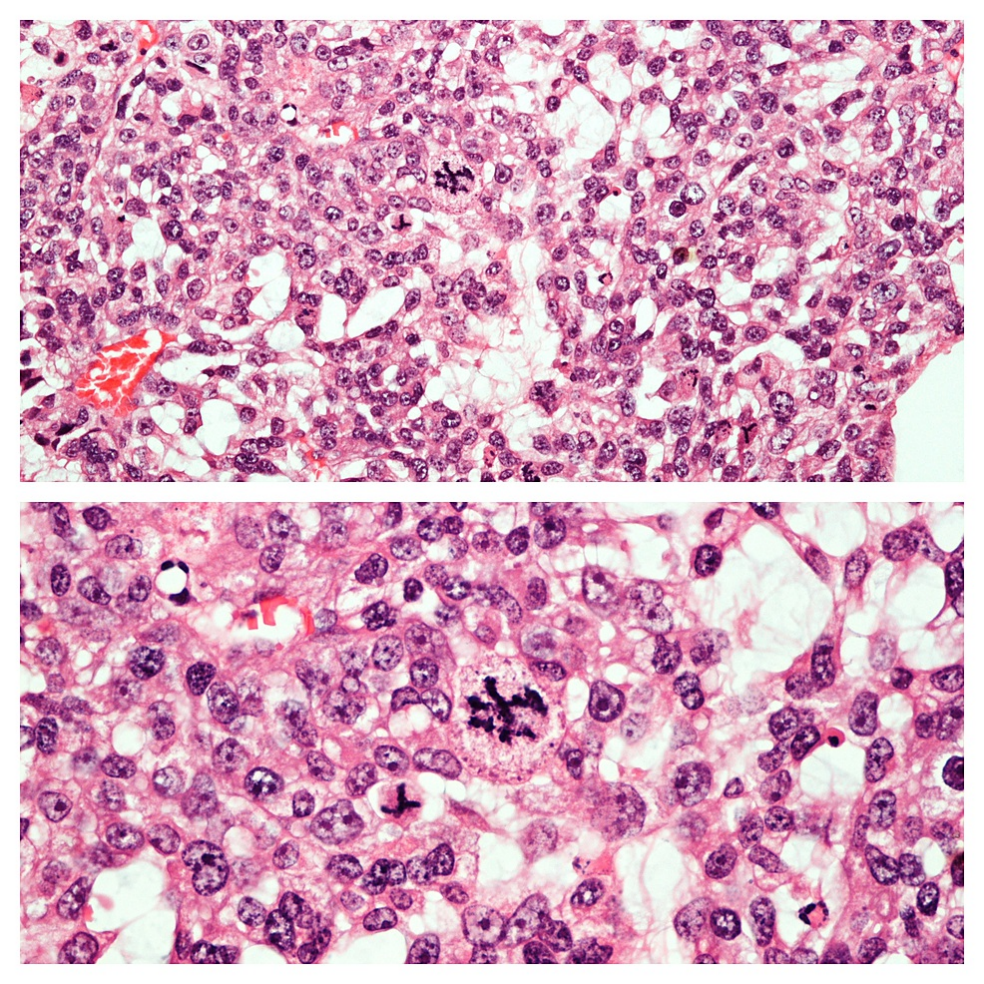
Histopathology of the excised lymph node. The upper half shows the area of the borderline phyllodes tumor and the lower half shows the area of the malignant phyllodes tumor in the excised lymph node (×100).

A 25-year-old lactating female with no comorbidities presented with a painless and fixed lump in her left breast. The patient noticed the sudden onset of the lump a year ago. The lump demonstrated a gradual increase in its size. The patient underwent physical examination by the breast surgeon that revealed a nontender and smooth mass in the lower outer quadrant of the left breast. The mass was soft to firm in consistency, demonstrated well-defined margins, and measured between 2 and 2.5 cm on palpation. On the basis of clinical history and physical examination, a provisional diagnosis of fibroadenoma was considered. The breast mass was excised and the patient did not undergo further investigations. Two months after the surgery, she presented with a recurrent lump on her left breast. A computed tomography (CT) with contrast was done for reassessment. CT showed regrowth of the mass in the lower outer quadrant of the left breast, with enlarged, matted, and necrotic ipsilateral axillary lymph nodes. She underwent wide local excision of the lump with ipsilateral axillary clearance in June 2020. Histopathology of the excised lump revealed a malignant PT with a maximum dimension of 2.5 cm (Figure 1). All margins of the excised lump were free of tumors; however, the involvement of the lymph nodes was positive demonstrating metastasis (Figure 2).

**Figure 3:**
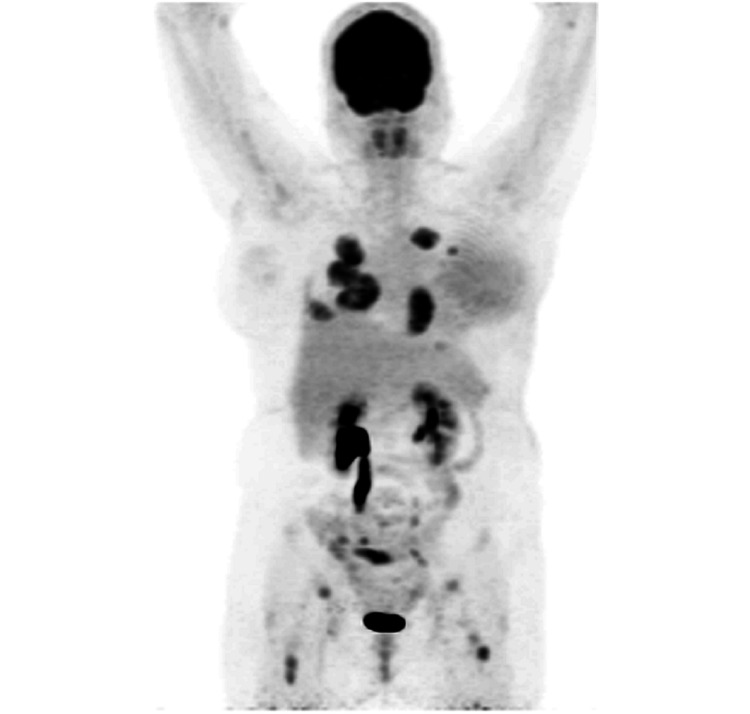
Maximum-intensity projection image of the FDG PET. Whole-body PET image showed multiple distant metastases. FDG: 18F-fluorodeoxyglucose; PET: positron emission tomography

Locoregional radiotherapy was administered in July 2020. Two months after the completion of the treatment, a follow-up CT scan revealed suspicious lung nodules. She was referred for restaging with FDG PET-CT (Figure 3).

**Figure 4:**
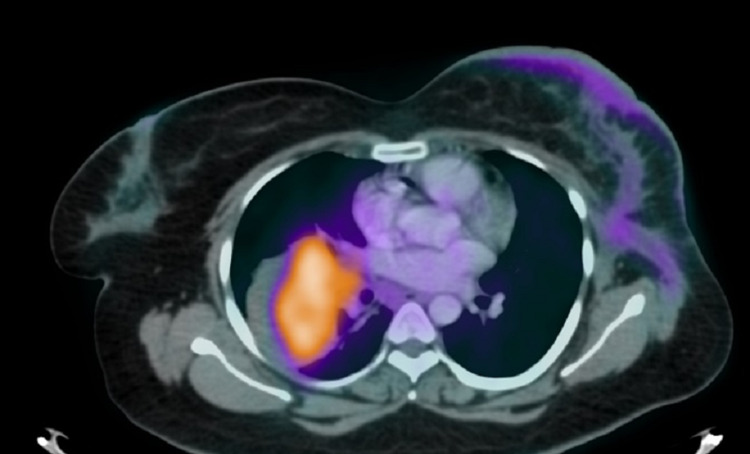
Fused axial slice of FDG PET-CT. A hypermetabolic right lung mass and mild accumulation of FDG in skin thickening overlying the left breast parenchyma. FDG: 18F-fluorodeoxyglucose; PET-CT: positron emission tomography-computed tomography

PET image (Figure 3) showed multiple hypermetabolic bilateral pulmonary masses, along with osseous and bone marrow metastases. Mildly avid cutaneous thickening was observed in the left breast, demonstrating a low standardized uptake value (SUVmax 1.7) (Figure 4).

**Figure 5:**
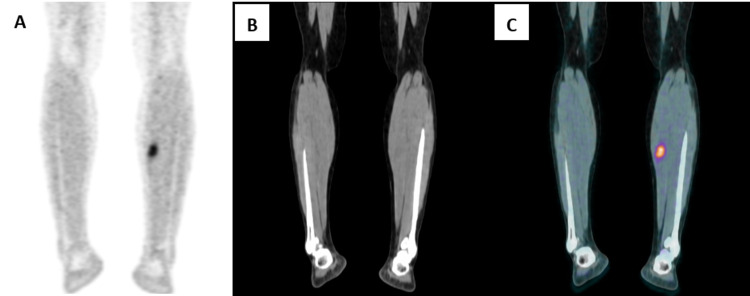
Coronal slices of the FDG PET-CT of the leg region. (A) PET images of the legs, revealing a hypermetabolic focus in the left leg. (B) CT component of the PET-CT showing a small hypodense lesion in the left soleus. (C) Fused PET-CT image showing intense FDG uptake in the left soleus corresponding to the hypodense lesion appreciated on the CT. CT: computed tomography; FDG: 18F-fluorodeoxyglucose; PET: positron emission tomography

The unique finding was a focal hypermetabolic and hypodense lesion in the left soleus (SUVmax 3.2) (Figure 5). The patient died due to extensive distant metastases over a short span of time.

## Discussion

Malignant PT is a rare neoplasm that can be clinically misdiagnosed as benign breast lesions, such as fibroadenoma. Its distinguishing characteristic is rapid growth. PT usually affects middle-aged women, with a mean age of 45 years [2]. PT may contain foci of benign, borderline, and malignant features intermixed within the same neoplasm. A malignant component is differentiated from borderline tumors by marked stromal cellularity, stromal overgrowth, nuclear pleomorphism, and high mitotic activity [2,8].

The estimated local recurrence and distant metastasis rates for PT are roughly 20% and 3.5%, respectively. While the local recurrence rate is as high as 40%, distant metastases, mostly to the liver, lungs, and bones, are observed in almost 27% of the cases [9]. A retrospective analysis of 295 patients [8] indicated that the five-year, disease-free survival rates were 96.9%, 83.3%, and 71.7% in patients with benign PT, borderline cases, and malignant subtypes, respectively. About 95% of deaths were related to malignant PT with distant metastases. The average survival in cases of metastasis was seven months (range: 2-17 months). These results show that metastatic PT is associated with a poor prognosis.

Atypical distant metastasis from malignant PT of the breast has been reported in the literature [3-6]. However, there are few isolated reports in the literature emphasizing the usefulness of FDG PET-CT in PT [6]. Literature review reveals limited case reports in which rare metastatic sites, such as skeletal muscle involvement, are observed. A mass lesion in the sternocleidomastoid detected using FDG PET-CT and confirmed to be the metastasis of invasive ductal breast carcinoma using a tru-cut biopsy has been reported in the literature [5].

Our case demonstrates the value of FDG PET-CT in detecting malignant PT as a reliable metabolic marker that can survey the whole body for the locoregional assessment of the disease and detection of distant metastases in a single examination. Additionally, a high metabolic activity depicted by high quantitative values (for example, SUVmax) of lesions reflects the aggressive behavior of the primary disease. Using FDG PET-CT in appropriate clinical settings can detect the disease at unsuspected sites and present an accurate and metabolically significant disease burden for which optimum therapy can be selected.

## Conclusions

Our case is unique in that extensive systemic metastasis developed in a very short duration of two to three months. FDG PET-CT detected metastatic deposits on the skeletal muscle in the calf, which is a very rare site for metastasis. Findings in our case also show that malignant PT is intensely FDG-avid. Thus, FDG PET-CT is useful in detecting PT and demonstrating rare unsuspected sites of metastasis. In conclusion, this is a rare case of malignant PT of the breast metastasizing to skeletal muscle demonstrating an aggressive course and poor prognosis.
